# Gold nanorods-conjugated TiO_2_ nanoclusters for the synergistic combination of phototherapeutic treatments of cancer cells

**DOI:** 10.1186/s12951-018-0432-4

**Published:** 2018-12-20

**Authors:** Jooran Lee, Young Hwa Lee, Chan Bae Jeong, Joon Sig Choi, Ki Soo Chang, Minjoong Yoon

**Affiliations:** 1 Division of Scientific Instrumentation, Korea Basic Science Institute, Daejeon, 34133 Republic of Korea; 2 Department of Biochemistry, College of Natural Sciences, Chungnam National University, Daejeon, 34134 Republic of Korea; 3 Medical Device Development Center, Osong Medical Innovation Foundation, Cheongju, Chungbuk 28160 Republic of Korea; 4 Department of Chemistry, College of Natural Sciences, Chungnam National University, Daejeon, 34134 Republic of Korea

**Keywords:** Phototherapeutic nanocomplexes, TiO_2_ nanoclusters, Gold nanorods, HeLa cells, Cancer therapy, Photodynamic therapy, Photothermal therapy

## Abstract

**Background:**

Recently, a combination of photodynamic therapy (PDT) and photothermal therapy (PTT) to generate reactive oxygen species (ROS) and heat to kill cancer cells, respectively has attracted considerable attention because it gives synergistic effects on the cancer treatment by utilizing the radiation of nontoxic low-energy photons such as long wavelength visible light and near IR (NIR) penetrating into subcutaneous region. For the effective combination of the phototherapies, various organic photosensitizer-conjugated gold nanocomplexes have been developed, but they have still some disadvantages due to photobleaching and unnecessary energy transfer of the organic photosensitizers.

**Results:**

In this study, we fabricated novel inorganic phototherapeutic nanocomplexes (Au NR–TiO_2_ NCs) by conjugating gold nanorods (Au NRs) with defective TiO_2_ nanoparticle clusters (*d*-TiO_2_ NP clusters) and characterized their optical and photothermal properties. They were observed to absorb a broad range of visible light and near IR (NIR) from 500 to 1000 nm, exhibiting the generation of ROS as well as the photothermal effect for the simultaneous application of PDT and PTT. The resultant combination of PDT and PTT treatments of HeLa cells incubated with the nanocomplexes caused a synergistic increase in the cell death compared to the single treatment.

**Conclusion:**

The higher efficacy of cell death by the combination of PDT and PTT treatments with the nanocomplexes is likely attributed to the increases of ROS generation from the TiO_2_ NCs with the aid of local surface plasma resonance (LSPR)-induced hot electrons and heat generation from Au NRs, suggesting that Au NR–TiO_2_ NCs are promising nanomaterials for the in vivo combinatorial phototherapy of cancer.

**Electronic supplementary material:**

The online version of this article (10.1186/s12951-018-0432-4) contains supplementary material, which is available to authorized users.

## Background

The most common types of cancer treatments [1–4] are chemotherapy, radiation therapy and/or surgery. However, such treatments have many well-known disadvantages, including relatively poor specificity toward malignant tissues, drug resistance and side effects [5, 6]. Therefore, there has been a demand for the development of the new treatment that can selectively eliminate only cancer cells/tissues without damage and side effects to normal cells/tissues. Recently, phototherapies including photodynamic therapy (PDT) and photothermal therapy (PTT) have received considerable attention as potential cancer therapies due to their advantages such as remote controllability, few complications, improved selectivity and rapid recovery [5, 7]. The phototherapy employs the photosensitizer (PS) or photothermal agent (PTA) that are nontoxic in the dark but able to selectively kill cancer cells by reactive oxygen species (ROS) or heat generated under the light irradiation without damage to normal tissues, respectively [8–11]. These photoreactions occur in the immediate locale of the light-absorbing PS or PTA which can be activated only in the particular areas of cancer cells/tissues that have been exposed to light. The most of the PSs used in cancer therapy are organic dyes such as porphyrin derivatives [7, 12–15], boron-dipyrromethene (BODIPY) conjugates [16] and methylene blue [17]. However, the PDT using the organic PSs gives unsatisfactory results because the PS’s absorb visible light mostly [18] with little absorption of near IR (NIR) (650–900 nm) penetrating deeply into biological tissue, and the PDT alone is not suitable for subcutaneous treatment. Thus, for the subcutaneous treatment, PTT using gold nanoparticles has been attracting interests because gold nanoparticles can absorb NIR radiation to generate heat killing cancer cells [11, 19–22]. Nevertheless, the efficacy of PTT is not so high as compared to that of PDT. Therefore, many researchers have attempted to apply the combination of PDT and PTT to enhance the therapeutic efficiency synergistically against malignant carcinomas as compared to PDT or PTT alone [10, 23–25].

For the effective combination of the phototherapies, many PS-conjugated PTA nanocomplexes have been developed. However, ROS generation by PS is more or less inhibited due to the excitation energy transfer from PS to PTA in addition to oxygen, and the combination efficiency cannot be maximized as anticipated. In order to overcome this problem, modification of the PS–PTA nanocomplexes has been performed by leaving the space between PS and PTA [7, 26–28]. Very recently Chung et al. [7] have prepared the dendrimer porphyrin (DP)-coated gold nanoshell (DP-AuNS) in which dendritic wedges of DP play a role as a spacer between porphyrin and PTA to minimize the additional energy transfer, and they found that the DP-AuNS could be applied to synergistic combination of the PDT and PTT. In spite of such improvements of PS–PTA nanocomplexes, they can’t be used for a long time because of photobleaching of organic photosensitizers. Thus, inorganic semiconductor nanomaterials would be rather useful as an alternative PS if they generate ROS. Among the semiconductor nanomaterials, TiO_2_ NPs are attracting much attention because of strong photocatalytic activity, non-toxicity, high photostability and inexpensiveness [29]. However, most of the pristine TiO_2_ NPs are active under UV light excitation which induces damage to biological components and its penetration into biological tissue is very limited to reach the cancer cells situated far away from the tissue surface. Thus, TiO_2_ NPs for in vivo treatment of subcutaneous cancers need to be modified to absorb long-wavelength visible light or NIR. Previously, we had synthesized defective TiO_2_ NPs (*d*-TiO_2_ NPs) which absorb a broad range of light from visible to NIR. The resultant *d*-TiO_2_ NPs were found to generate ROS including singlet oxygen (^1^O_2_) by a different mechanism other than the excitation energy transfer under the long wavelength visible light irradiation [18], leading to killing cancer cells by PDT pathway.

Hereby, as a new nanocomplex for the effective combination of PDT and PTT, we fabricated gold nanorods (Au NRs) conjugated with *d*-TiO_2_ NP clusters (Au NR–TiO_2_ NCs) by functionalizing with (3-aminopropyl) triethoxysilane (APTES) [30, 31] and polyethylene glycol (PEG) [32–36]. Their optical and photothermal properties were characterized, supporting that they generate ROS and heat upon irradiation of long wavelength visible light and NIR, respectively. Thus, the simultaneous application of PDT and PTT of cancer cells (HeLa cells) incubated with the nanocomplexes exhibited a synergistic increase of cell death by the enhanced generation of ROS from TiO_2_ NPs with the aid of the NIR-induced heat from the Au NRs.

## Results

### Preparation and characterization of Au NR–TiO_2_ NCs

**Fig. 1:**
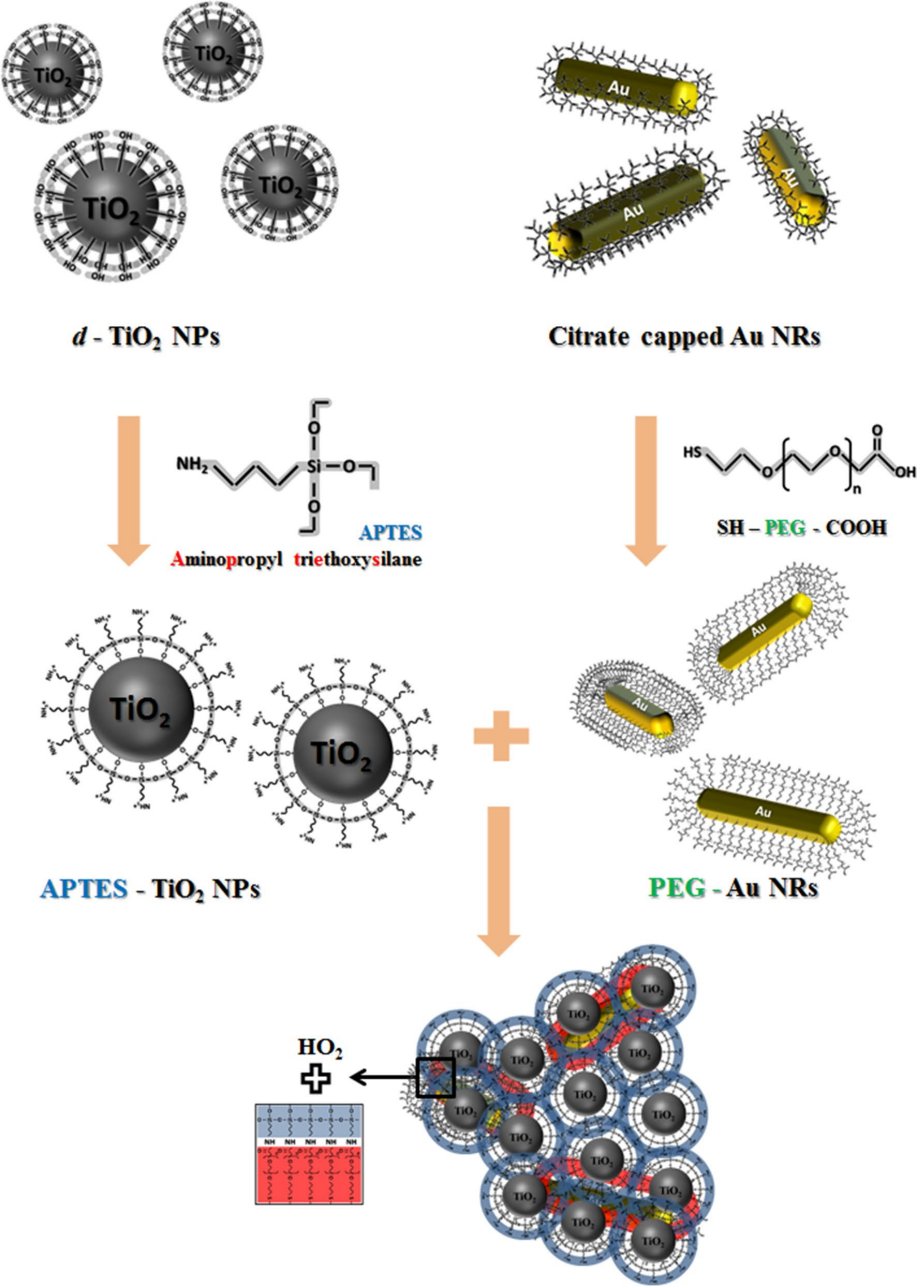
Schematic illustration of the preparation of Au NR–TiO_2_ NCs

The fabrication processes of new photofunctional nanocomplexes for the combination of phototherapeutic treatments are illustrated in Fig. 1. Firstly, *d*-TiO_2_ NPs were synthesized by hydrothermal reaction of liposome-TiO_2_ composites as previously reported [18], and their surfaces were modified by binding with APTES. In parallel with this procedure, the citrates capped on the surfaces of Au NRs were exchanged with HS–PEG–COOH. Then, preparation of the Au NR–TiO_2_ nanocomplexes (Au NR–TiO_2_ NCs) was performed by coupling of the two surface-modified nanomaterials to lead the formation of the amide bond between the amine group of APTES–TiO_2_ NPs and the carboxyl group of PEG–Au NRs [37, 38].

**Fig. 2:**
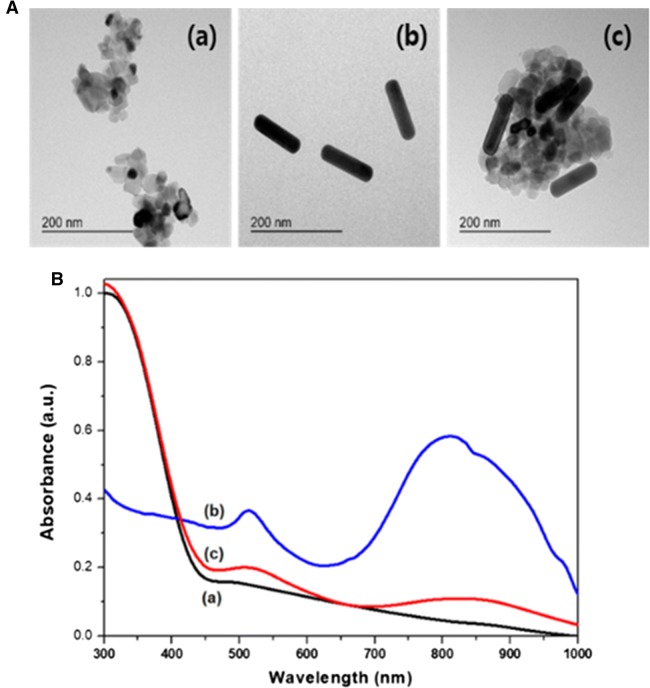
**A** TEM images and **B** UV–visible reflectance absorption spectra of APTES–TiO_2_ NPs (a), PEG–Au NRs (b) and Au NR–TiO_2_ NCs (c)

Figure 2A (a) and (b) show the TEM images of the as-prepared APTES–TiO_2_ NPs (average diameter of ~ 25 nm) and PEG–Au NRs (average aspect ratio of ~ 4, 100 nm length and 25 nm diameter), respectively. In fact, APTES–TiO_2_ NPs in aqueous solution was observed to agglomerate together to form clusters. The average diameter of a cluster was evaluated to be about 224.2 nm by dynamic light scattering (DLS) measurement (Additional file 1: Figure S1). The cluster size was observed to be increased by about 90 nm after mixing with PEG–Au NRs as shown in Fig. 2A (c), indicating the formation of the Au NR–TiO_2_ NCs by coordinating between APTES and PEG. The formation of the Au NR–TiO_2_ NCs was also confirmed with the diffuse reflectance UV–visible absorbance measurement at room temperature. APTES–TiO_2_ NPs exhibited a broad visible light absorption band (500–800 nm) in addition to UV absorption band [Fig. 2B (a)] due to the surface defects as previously reported [7], and PEG–Au NRs exhibited two localized surface plasma resonance (LSPR) absorption peaks at 510 nm and 800 nm. The LSPR absorption peaks shifted to longer wavelengths at 525 nm and 850 nm upon mixing PEG–Au NRs with APTES–TiO_2_ NPs. This supports that the Au NRs are conjugated with *d*-TiO_2_ NPs, and the final product of Au NR–TiO_2_ NCs could absorb broad visible light as well as NIR light. When Au NR–TiO_2_ NCs were dispersed in deionized water at pH 7.0 at a concentration of 1 mg mL^−1^, they were found to have an average diameter of 317 nm which is appropriate size for their efficient uptake into cells as is within the known range from about 50 to several hundred nanometers (Additional file 1: Figure S1) [39].

**Fig. 3:**
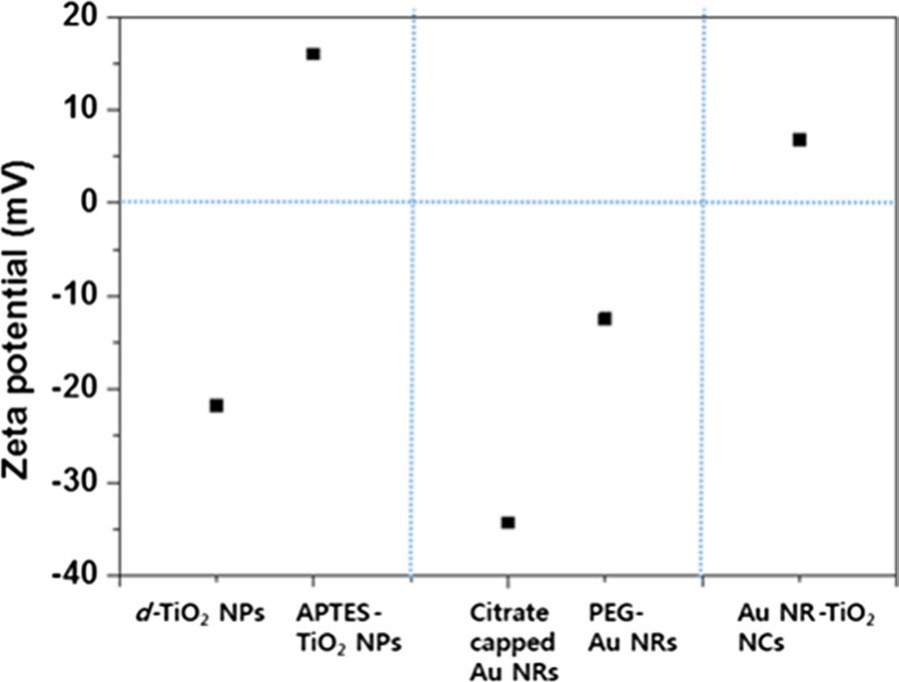
The change of the zeta potential of the TiO_2_ NPs and Au NRs after conjugation with different molecules

In addition to the size of nanocomplexes their surface charges are also known to be an important factor influencing the cell uptake efficiency. Generally, positively charged particles are taken up better than negatively charged ones because the cell plasma membrane is negatively charged [40–42]. In order to estimate the surface charges, the ζ-potentials of different nanoparticles including Au NR–TiO_2_ NCs were measured (Fig. 3 and Additional file 1: Figure S2). As shown in Additional file 1: Figure S2, the average ζ-potential of *d*-TiO_2_ NPs in deionized water at pH 7.0 was − 21.7 mV, indicating that the surfaces of *d*-TiO_2_ NPs are negatively charged. However, the ζ-potential of APTES–TiO_2_ NPs was significantly changed to + 16.0 mV. This may be due to ionization of –NH_2_ groups of APTES to –NH_3_. On the other hand, the ζ-potential of Au NRs was − 12.5 mV even after surface modification with PEG, but this negative potential was observed to be changed to a positive value of + 6.8 mV upon mixing with APTES–TiO_2_ NPs, supporting again that *d*-TiO_2_ NPs are bonded to the Au NRs. Therefore, the as-synthesized Au NR–TiO_2_ NCs are anticipated to be easily taken up to the cellular membranes and entered into the cytoplasm probably via endocytosis [43].

### Intracellular generation of ROS from nanoparticles

**Fig. 4:**
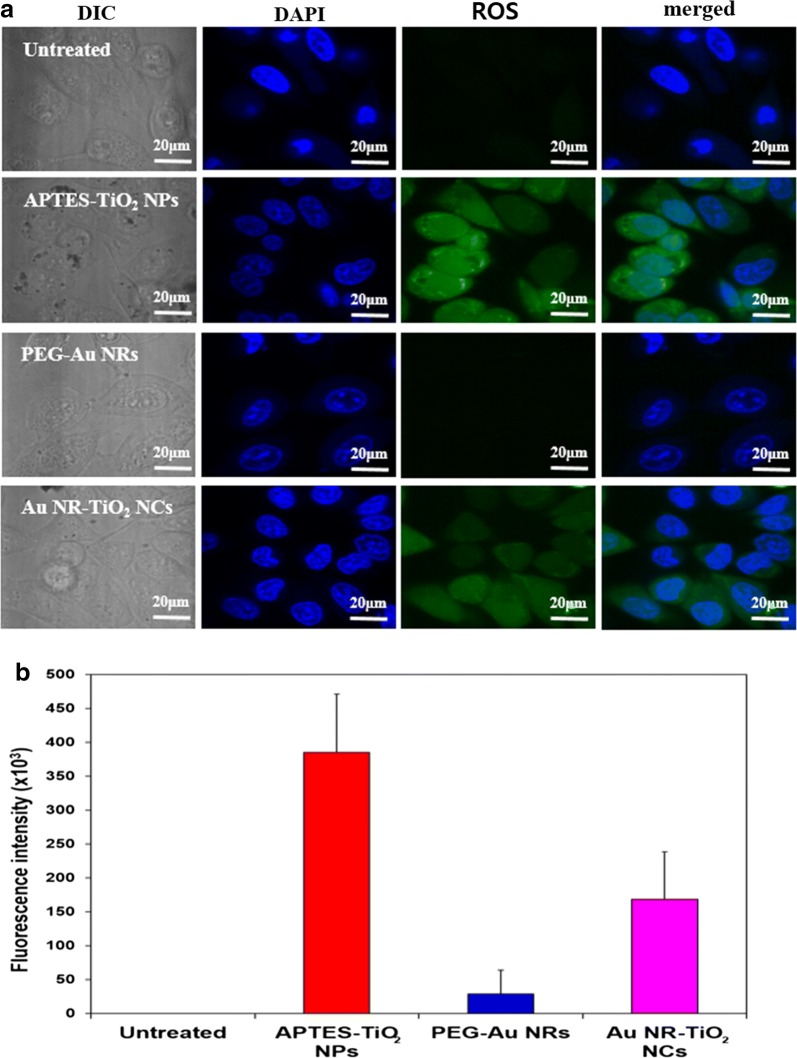
**a** Laser scanning confocal microscopy images of HeLa cells incubated with nanoparticles; DIC images, blue fluorescence images after staining with DAPI and green fluorescence of ROS probe (H_2_DCFDA) under the visible light irradiation. **b** Quantification of the generated ROS using fluorescence intensity

To evaluate the generation of intracellular generation of ROS from the nanoparticles in cancer cells, firstly HeLa cells were incubated with 100 μg/mL of three different nanoparticles (APTES–TiO_2_ NPs, PEG–Au NRs, and Au NR–TiO_2_ NCs) and their confocal images were measured. Figure 4a shows the confocal DIC images which were compared with blue fluorescent images of DAPI (4′,6-diamidino-2-phenylindole), a nucleus stain of HeLa. Comparison of these images revealed that cytoplasm of the cells treated with APTES–TiO_2_ NPs or Au NR–TiO_2_ NCs exhibited dark spots in contrast to clear cytoplasm of the untreated or PEG–Au NRs, indicating that PEG–Au NRs are not internalized into the cells whereas APTES–TiO_2_ NPs or Au NR–TiO_2_ NCs are easily entered into the cells by endocytosis [44]. This is consistent with the fact that APTES–TiO_2_ NPs or Au NR–TiO_2_ NCs have positive ζ-potential for the efficient cell uptake while PEG–Au NRs have negative ζ-potential as aforementioned. It is also noteworthy that similar cell morphology was retained in the dark whether or not treated with nanoparticles, indicating no major effect of nanoparticles themselves on cell viability (Additional file 1: Figure S3 (A)).

The intracellular ROS generation was examined by monitoring green fluorescence from a standard ROS probe [40], H_2_DCFDA upon visible light irradiation. A strong green fluorescence was observed in the cytoplasm of Hela cells treated with APTES–TiO_2_ NPs and Au NR–TiO_2_ NCs while not observed in untreated cells, indicating that the nanoparticles were taken up and internalized to generate ROS upon irradiation. Quantification of ROS based on fluorescence intensities (Fig. 4b) revealed that the significantly high level of ROS was produced by APTES–TiO_2_ NPs and Au NR–TiO_2_ NCs as compared with negligible level generated by PEG–Au NRs. It is noteworthy that such green fluorescence was not observed from any nanoparticles-treated cells upon irradiation with NIR at 808 nm [Additional file 1: Figure S3 (B)]. These results demonstrate that the observed ROS generation in the cells was resulted from the absorption of the visible light by TiO_2_ NPs. Therefore, the relatively lower level of ROS produced from Au NR–TiO_2_ NCs is attributed to inhibition of directly visible light absorption of NPs by the LSPR of Au NRs or the LSPR-enhanced charge transfer quenching of the visible-light-induced electrons which are supposed to produce ROS [45]. Anyhow, it is evident that Au NR–TiO_2_ NCs are able to not only generate the significant amount of ROS but also induce photothermal heating from Au NRs exposed to NIR light which is caused by electron energy loss due to its longitudinal LSPR oscillation [46]. Thus, it would be worthwhile to attempt to combine both photodynamic and photothermal effects with Au NR–TiO_2_ NCs using visible light and NIR.

### Photothermal properties of nanoparticles and nanocomplexes

**Fig. 5:**
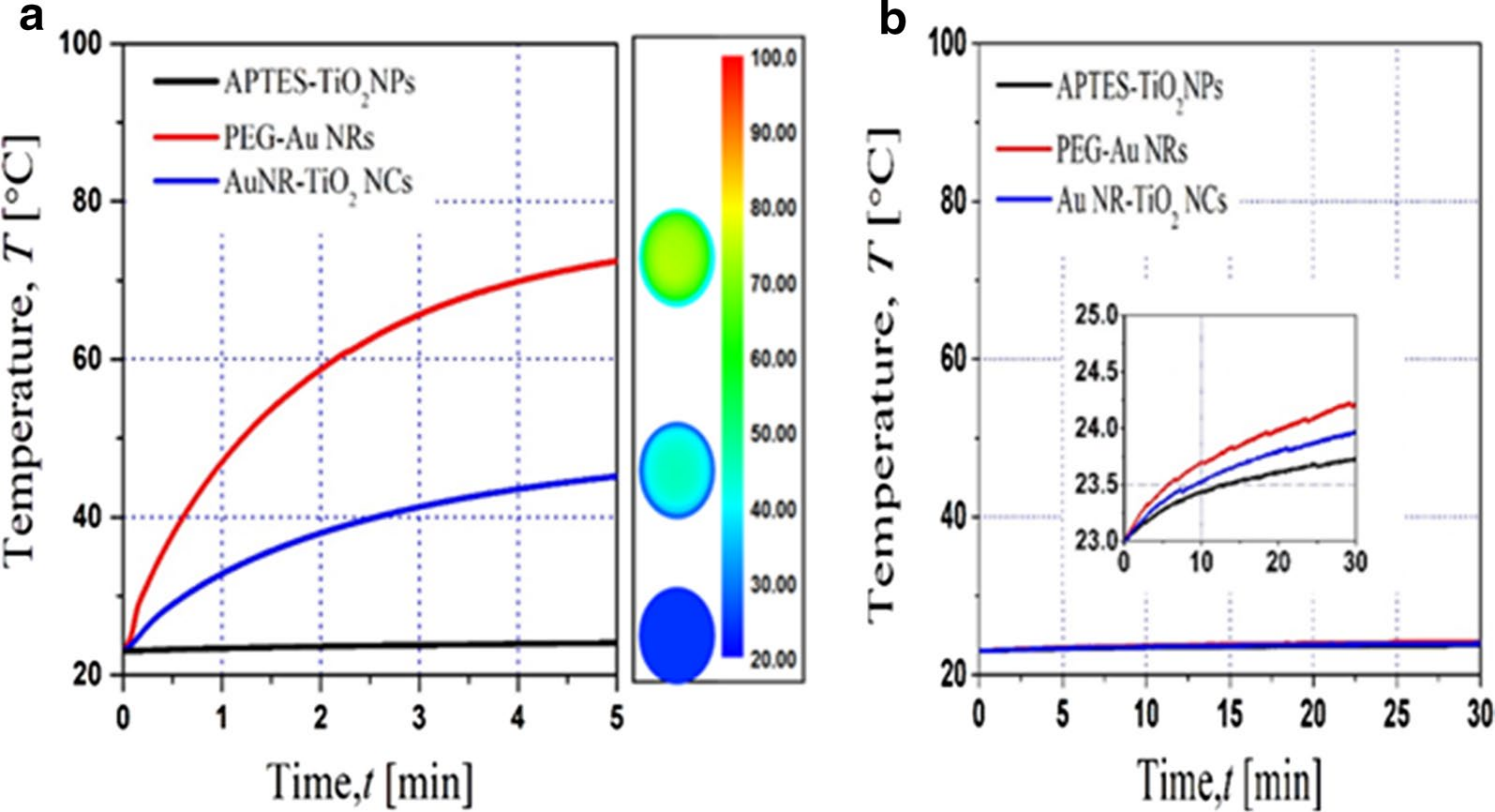
Temperature changes of the cell-culture solution containing different nano-particles (100 μg mL^−1^) as a function of time after NIR (808 nm laser) (**a**) and visible light exposure (**b**)

In order to examine the photothermal heating in the nanoparticles (nanocomplexes)-containing HeLa cells, the temperature of the cell culture solution was measured with infrared camera upon irradiation using an 808 nm NIR laser (1 W/cm^2^) and a xenon lamp. Figure 5A shows the temperature changes of the solution containing 100 μg mL^−1^ of three different nanoparticles such as APTES**–**TiO_2_ NPs, PEG**–**Au NRs and Au NR–TiO_2_ NCs as a function of NIR irradiation time. The highest temperature was reached up to 72 °C from 23 °C rapidly 5 min after irradiation on PEG**–**Au NRs and the medium temperature change was observed up to 45 °C with Au NR**–**TiO_2_ NCs in contrast to negligible temperature change with APTES**–**TiO_2_ NPs. However, such temperature changes were not significantly observed upon irradiation with visible light (Fig. 5b). These results indicate that the photothermal heating is induced by Au NRs in Au NR-TiO_2_ NCs upon exposure to NIR. Such photothermal heating could be observed even by using the small amount of Au NR**–**TiO_2_ NCs (Additional file 1: Figure S4).

### In vitro cell viability under the dark and light irradiation conditions

**Fig. 6:**
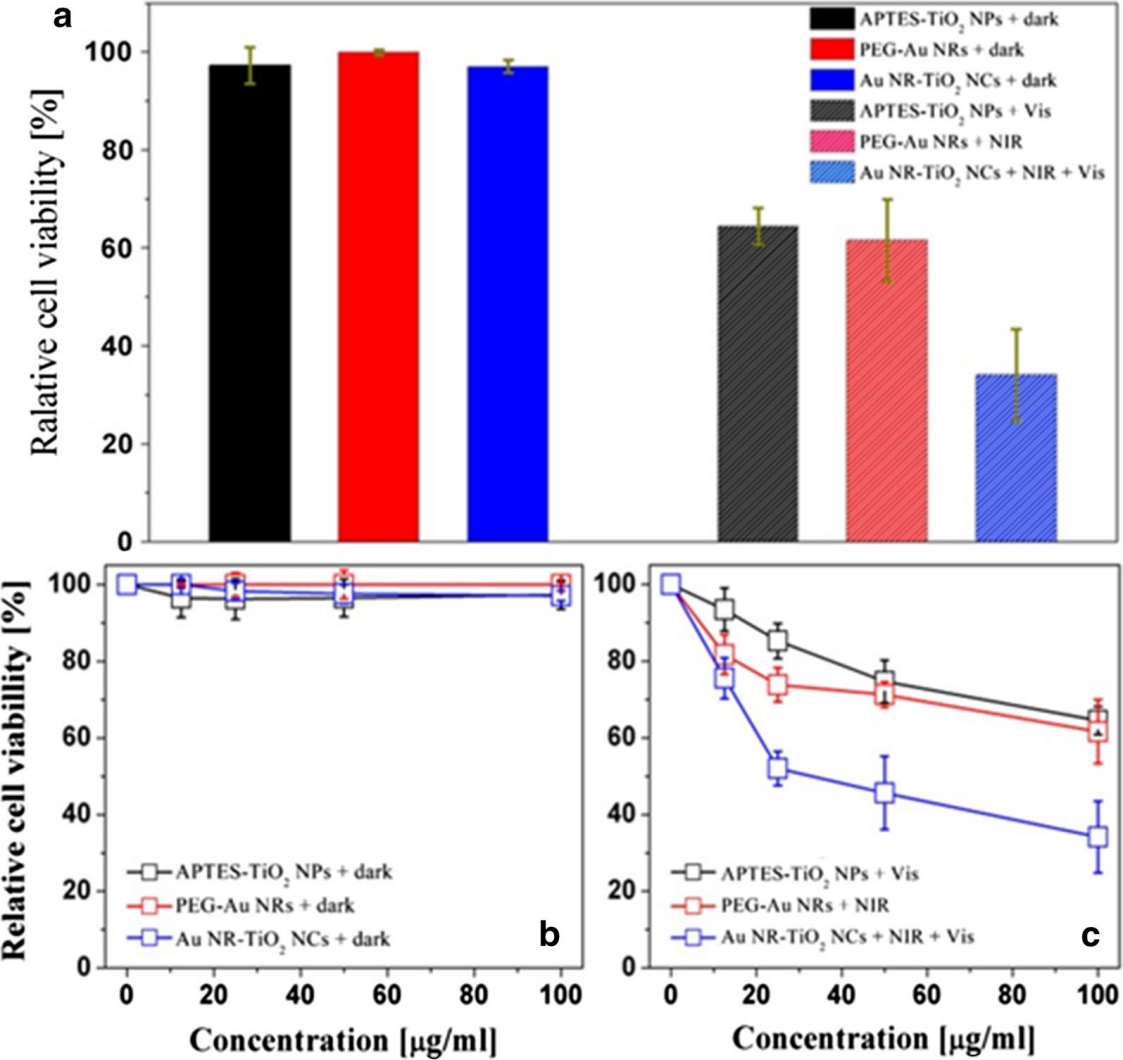
Relative cell viabilities of HeLa cells incubated with various concentrations of APTES–TiO_2_ NPs, PEG–Au NRs and Au NR–TiO_2_ NCs: Histogram of the effects of the nanoparticles (100 μg mL^−1^) on the relative viabilities of HeLa cells under the dark (left) and photoirradiation (right) conditions (**a**), Relative cell viabilities as a function of nanoparticle concentration under the dark condition (**b**) and photoirradiation conditions (**c**). Note: The photoirradiation was performed by sequential irradiation of visible light for 30 min and NIR for 5 min. The viabilities of the cells without nanoparticles were confirmed to be unaffected by the same photoirradiation condition

The viability of HeLa cells with different treatments was estimated by evaluating their cytotoxicity effect on Hela cells using the EZ-Cytox reagent based on the water-soluble tetrazolium (WST) method. For the control experiments, all the cells were incubated with nanoparticles such as APTES–TiO_2_ NPs, PEG–Au NRs, and Au NR–TiO_2_ NCs under the dark condition for 2 days, and they were observed to survive almost completely like the untreated cells. Figure 6a (left) demonstrates that all the nanoparticles showed good cytocompatibility (> 98%) over various concentrations from 10 μg mL^−1^ up to 100 μg mL^−1^ in dark (Fig. 6b). In contrast to the high cell viability under the dark condition, the HeLa cells incubated with 100 μg mL^−1^ of APTES–TiO_2_ NPs, PEG–Au NRs or Au NR–TiO_2_ NCs exhibited extensive cell death upon photoirradiation as shown in Fig. 6a (right), even though the viabilities of the cells without nanoparticles were not affected by the sequential irradiation of visible light for 30 min and NIR for 5 min. As shown in Fig. 6c, the photoinduced cell death was observed to depend on the concentrations of nanoparticles or nanocomplexes under different photoirradiation conditions, starting even with the low dose (10 μg mL^−1)^ and further increase with the concentration increased to 100 μg mL^−1^, indicating that all the three nanoparticles photocatalyzed the killing of cancer cells under the photoirradiation.

## Discussion

**Fig. 7:**
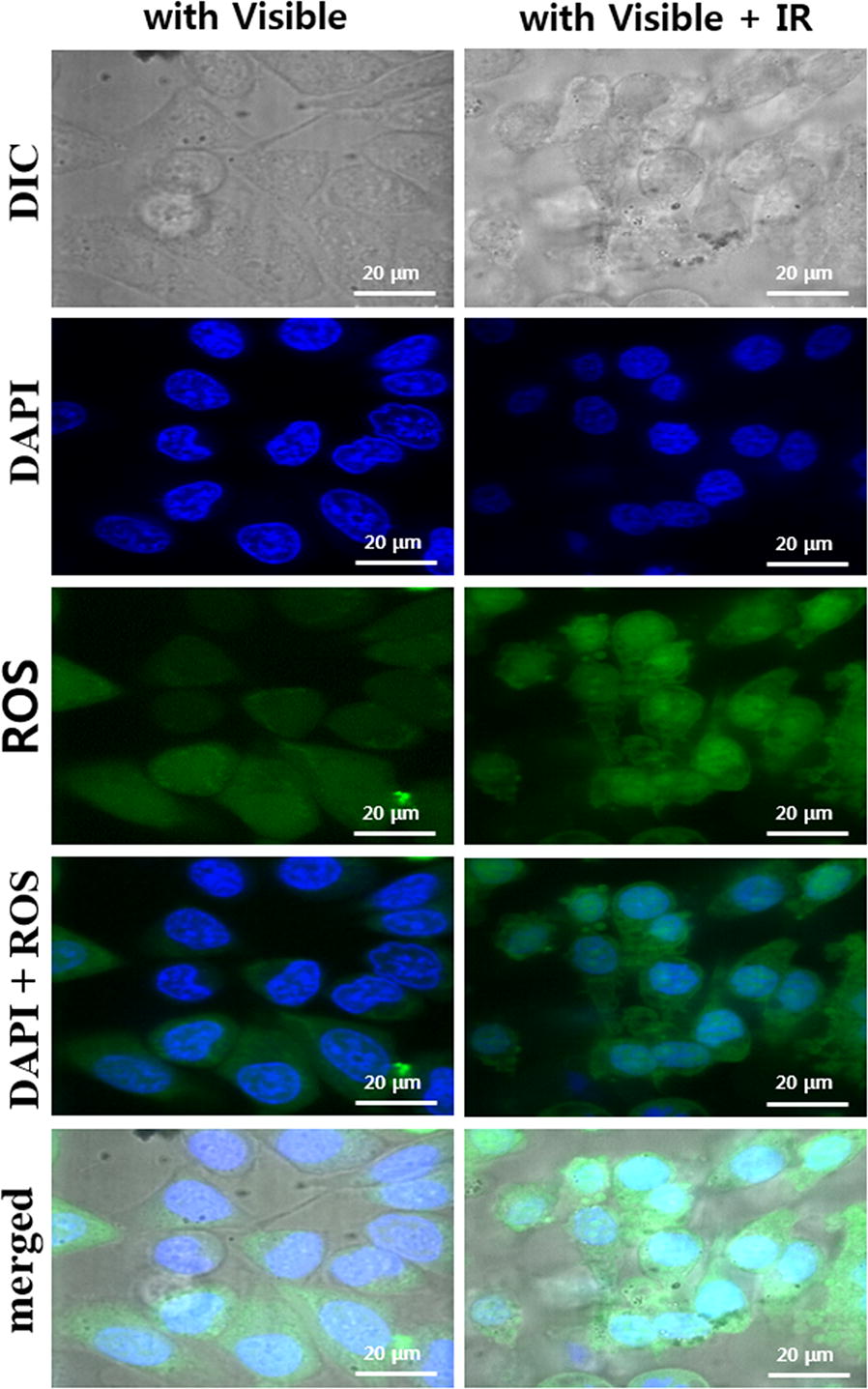
Laser scanning confocal microscopy images of HeLa cells incubated with Au NR–TiO_2_ NCs irradated with visible light or combined light (visible light and NIR): DIC images, blue fluorescence images after staining with DAPI and green fluorescence images indicating ROS generation with under visible light or visible light combined NIR (808 nm laser) irradiation

The observed cell death with APTES**–**TiO_2_ NPs or PEG**–**Au NRs was caused by single irradiation of visible light or NIR respectively, suggesting that APTES**–**TiO_2_ NPs or PEG**–**Au NRs make the most of PDT or PTT effects separately as the maximum ROS or heat was generated from APTES**–**TiO_2_ NPs or PEG**–**Au NRs, respectively under the single irradiation of visible light or NIR (Additional file 1: Figure S4 and Fig. 5). Nevertheless, their efficacy of the photoinduced cell death was observed to be similar and low with about 20–30% killing at a concentration of 100 μg mL^−1^. On the other hand, about two times higher cell death (30% cell viability) was achieved from the cells incubated with the same concentration of the nanocomplex, Au NR**–**TiO_2_ NCs upon simultaneous irradiation of visible light and NIR using the irradiation set up shown in Additional file 1: Figure S5, even though ROS or heat generated from Au NR–TiO_2_ NCs upon single irradiation of visible light or NIR is rather two times lower than those from APTES–TiO_2_ NPs or PEG–Au NRs (Figs. 4 and 5). Particularly it should be noted that from the confocal microscopic images of the cells incubated with Au NR–TiO_2_ NCs (Fig. 7) much stronger green fluorescence was observed to emit as a result of the simultaneous irradiation rather than the visible light irradiation alone. These results imply that the higher efficacy of cell death in the presence of Au NR–TiO_2_ NCs is likely due to the visible-light-induced ROS generation enhanced synergistically by the heat generated by NIR irradiation, considering that the temperature-dependent (up to 40 °C) increases of ROS generation had been observed in the process of the aminolaevulinic acid (ALA)-treated PDT of human skin fibroblasts [47].

**Fig. 8:**
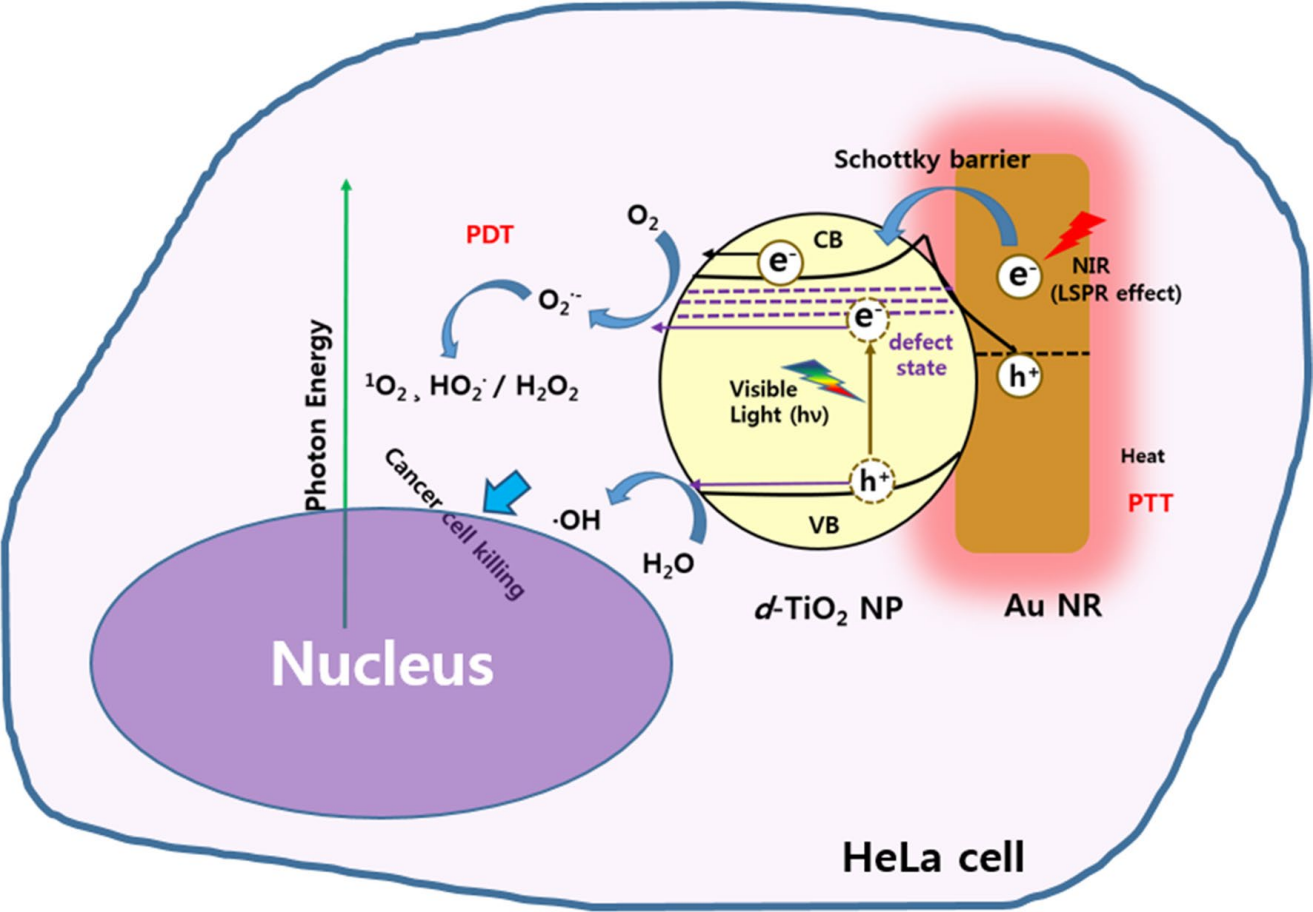
The proposed mechanism of synergistic generation of ROS by simultaneous irradiation with visible light and NIR to kill cancer cells

Thus, the mechanism of the photokilling of cancer cells with Au NR–TiO_2_ NCs can be proposed as illustrated in Fig. 8. When both visible light (420–780 nm) and NIR are irradiated on Au NR–TiO_2_ NCs, the visible and NIR photons are absorbed by *d*-TiO_2_ NP and Au NR, respectively. The visible light energy separate electrons and holes in the defective states and the valence band (VB) of *d*-TiO_2_ NPs, respectively. The electrons in the defective states can react with oxygen molecules in the solution to produce a certain amount of superoxide radicals (^·^O_2_^−^) as one of ROS to be used for the cell killing while the photogenerated holes in VB can react with H_2_O to form hydroxyl radicals (^·^OH) as another type of ROS. On the other hand, the NIR irradiation causes LSPR excitation of Au NRs are known to form hot electrons which are injected into the conduction band (CB) of the conjugated TiO_2_ NPs with photogenerated holes left in Au NRs [48, 49]. Such an interfacial electron transfer from Au to TiO_2_ in the nanocomplex would be further facilitated by overcoming Schottky barrier through the NIR-induced heat generation from the Au NRs as reported in other Au–TiO_2_ nanohybrids which are applied to visible light photocatalysts for the destruction of pollutants [50–53]. Consequently, more ^·^O_2_^−^ can be produced by the reaction of oxygen molecules with the injected electrons in the CB of TiO_2_ NPs through the LSPR excitation. Also, the holes in Au NRs also produce additional ^·^OH. Therefore, we can conclude that the simultaneous irradiation with visible light and NIR on Au NR–TiO_2_ NCs produce ROS synergistically from *d*-TiO_2_ NPs with the aid of LSPR-induced electron and heat generation from the conjugated Au–NRs, and we believe that the nanocomplex Au NR–TiO_2_ NCs are a greatly potent phototherapeutic nanomaterials for the improved application of combination of PDT and PTT to the cancer therapy.

## Conclusion

Significant PDT and PTT effects were observed from HeLa cells treated with *d*-TiO_2_ NPs or Au NRs upon visible light or NIR irradiation alone. Further synergistic enhancement of the phototherapeutic efficiency was achieved by simultaneous irradiation with visible light and NIR onto the cells incubated with inorganic nanocomplex such as Au NR–TiO_2_ NCs which were newly fabricated by coupling *d*-TiO_2_ NPs and Au NRs. It was found that visible light-induced ROS production from the Au NR–TiO_2_ NCs increased with the aid of LSPR-induced hot electrons and heat generation. Therefore the combination of PDT and PTT treatments with Au NR–TiO_2_ NCs has a great potential to be applied to improve the cancer therapy.

### Materials and experimental methods

#### Chemicals

All of the chemicals used for the synthesis of AuNR–TiO_2_NPs, including P-25 (Degussa-Huls), egg lecithin (L-R-phosphatidylcholine, > 60%, Sigma-Aldrich), chloroform (> 99.8%, Samchun, Korea), sodium hydroxide (> 93%, Duksan, Korea), ethanol (200–proof, > 99.8%, Sigma-Aldrich), (3-aminopropyl)triethoxysilane (> 99%, Sigma-Aldrich), citrates-capped Au NRs (AC12-25-808-CIT-DIH-1, Nanopartz), HS–PEG–COOH (MW 10 k, Nanocs), EDC (*N*-(3-dimethylaminopropyl)-*N*′-ethylcarbodiimide hydrochloride, > 98%, Sigma-Aldrich) and NHS (*N*-hydroxysuccinimide, > 98%, Sigma-Aldrich) were of analytical grade and used as purchased without further purification.

#### Synthesis of Au NR–TiO_2_ NCs

Firstly, the surfaces of as-prepared *d*-TiO_2_ NPs were modified by binding with APTES as per the previous report [18]. In the first step, APTES solution was prepared by dissolving 0.1% acetic acid, 4% deionized water and 2% APTES in ethanol. The *d*-TiO_2_ NPs were dispersed in a beaker containing 10 mL ethanol under constant ultrasonication for 30 min. Then, the APTES solution was added into the ethanol dispersed *d*-TiO_2_ NPs solution and stirred for 24 h. The solution was washed with ethanol and deionized water several times by repeated centrifugation. An aliquot of these particles was dried in an oven at 40 °C. Next, the purchased citrates-capped Au NRs were exchanged with HS–PEG–COOH to prepare the PEG–Au NRs. Finally, the preparation of the Au NR–TiO_2_ NCs was performed by coupling of the two surface-modified nanomaterials (APTES–TiO_2_ NPs and PEG–Au NRs) to lead to the formation of the amide bond between the amine group of APTES and the carboxyl group of PEG [37, 38].

#### Structural and optical characterization of nanoparticles

The morphology of the as-prepared *d*-TiO_2_ NPs, PEG–Au NRs and Au NR–TiO_2_ NCs were examined by transmission electron microscopy (TEM; Tecnai G2 F30). The TEM sample was prepared by dip-coating Formvar/carbon film-Cu grids with a nanocolloidal solution obtained by sonication of the synthesized nanoparticles in ethanol.

The particle sizes and ζ-potentials of the dispersed solution of the synthesized nanoparticles were measured respectively by dynamic light scattering (DLS) and laser Doppler velocimetry (LDV) using an electrophoretic light scattering spectrophotometer (Otsuka Electronics Co., Ltd; ELS-Z2).

For the optical properties, diffuse reflectance UV–VIS-NIR absorption spectra (DRS) were recorded using a Solid Spec-3700 double beam spectrophotometer equipped with an integrating sphere.

#### Cell culture and imaging

The human cervical carcinoma cells (HeLa) were grown in 89% Dulbecco’s modified Eagle’s medium with 10% fetal bovine serum and 1% antibiotic–antimycotic solution. The cells were routinely maintained in the plastic tissue culture dishes at 37 °C under a humidified 5% CO_2_—containing an atmosphere.

The cell images were obtained by observing differential interference contrast (DIC) and fluorescence of DAPI (4′,6-diamidino-2-phenylindole) or H_2_DCFDA using laser-scanning confocal microscopy (LSCM; LSM5 live configuration Vario Two VRGB).

#### Measurement of ROS generation under visible region

For the measurement of ROS generation, HeLa cells were seeded in a 15 μ-slide 8 well plate (Ibidi, Germany) at a density of 5 × 10^3^ cells per well and incubated for 24 h at 37 °C under a 5% CO_2_ atmosphere followed by addition of 10 μg/mL of APTES–TiO_2_ NPs, PEG–Au NRs or Au NR–TiO_2_ NCs. After 24 h, the culture medium was replaced by the new medium contained 20 μg/mL carboxy-H_2_DCFDA (Sigma-Aldrich) for 30 min in the conventional incubator (37 °C, 5% CO_2_). Next, carboxy-H_2_DCFDA-containing medium was removed and added the fresh medium. Then, the 8 well plates were irradiated under broadband visible-light region (12 mW/cm^2^) with a xenon lamp (Asahi Spectra, MAX-302, Japan) for 30 min. Subsequently, the nucleus was stained with 10 μg/mL bisbenzimide trihydrochloride (Hoechest33342) for 10 min and washed with DPBS several times. Fluorescence images of the intracellular ROS generation detected by carboxy-H_2_DCFDA D were obtained with a confocal laser microscope (Zeiss LSM5 live configuration Vario two VRGB).

#### Photothermal properties of nanoparticles

To measure the photothermal conversion performance of APTES–TiO_2_ NPs, PEG–Au NRs and Au NR–TiO_2_ NCs, temperature of the nanoparticles-containing solutions were measured with infrared camera (640 × 512 cooled InSb IRFPA with 90 µm pixel pitch) after irradiation using a 808 nm NIR laser (1 W/cm^2^) or broadband visible light from a xenon lamp (12 mW/cm^2^).

#### Evaluation of cytotoxicity and cell viability

The cytotoxicity of the injected nanoparticles was evaluated using the EZ-Cytox reagent (Daeil Lab Service, Seoul, South Korea) based on the water-soluble tetrazolium (WST) method. HeLa cells were seeded at a density of 1 × 10^4^ cells per well in a 96-well microassay plate and incubated for 24 h at 37 °C under a 5% CO_2_ atmosphere. The APTES–TiO_2_ NPs, PEG–Au NRs or Au NR–TiO_2_NCs were added to the incubated cells at various concentrations, followed by further incubation for an additional 24 h at 37 °C. Next, 10 μL of the EZ-Cytox reagent was added and the plates were incubated for 2 h at 37 °C. The absorbance of the EZ-Cytox reagent was measured at 450 nm using a microplate reader (VersaMax, Molecular Devices, USA). The cell viability (%) was calculated using the following equation: cell viability (%) = (OD_450(sample)_/OD_450(control)_) × 100. The control condition was maintained with cells not treated with anything, neither nanoparticles nor visible/NIR light.

#### Photoirradiation method

The HeLa cells incubated with APTES–TiO_2_ NPs, PEG–Au NRs or Au NR–TiO_2_ NCs were irradiated for 30 min with the visible light (12 mW/cm^2^) emitted from a xenon lamp (Asahi Spectra, MAX-302, Japan) or/and 5 min with an 808 nm NIR laser (1 W/cm^2^) (see Additional file 1: Figure S5).

## Additional file

**Additional file 1: Figure S1.** Sizes of APTES-TiO_2_ NPs (A), PEG-Au NRs (B) and Au NR-TiO_2_ NCs (C) were measured in deionized water at pH 7.0 by dynamic light scattering (DLS). **Figure S2.** Surface charge or zeta potential of TiO_2_ NPs (A), Au NRs (B) and Au NR-TiO_2_ NCs (C) were measured in deionized water at pH 7.0 by laser doppler velocimetry (LDV). **Figure S3.** Laser scanning confocal microscopy images of HeLa cells. DIC images and fluorescence images indicating ROS generation with nanoparticles in the dark (A) or under NIR (808 nm laser) light irradiation (B). **Figure S4.** Temperature changes of the cell-culture solutions containing various concentrations of APTES-TiO_2_ NPs (A), PEG-Au NRs (B) and Au NR-TiO_2_ NCs (C) with various as a function of NIR (808 nm laser) exposure time. **Figure S5.** Schematic illustration of the optical system for light irradiation.

## References

[1] Zou L, Wang H, He B, Zeng L, Tan T, Cao H, He X, Zhang Z, Guo S, Li Y (2016). Current approaches of photothermal therapy in treating cancer metastasis with nanotherapeutics. Theranostics.

[2] Eckhardt BL, Francis PA, Parker BS, Anderson RL (2012). Strategies for the discovery and development of therapies for metastatic breast cancer. Nat Rev Drug Discov.

[3] Schroeder A, Heller DA, Winslow MM, Dahlman JE, Partt GW, Langer R, Jacks T, Anderson DG (2011). Treating metastatic cancer with nanotechnology. Nat Rev Cancer.

[4] Owonikoko TK, Arbiser J, Zelnak A, Shu HK, Shim H, Robin AM, Kalkanis SN, Whitsett TG, Salhia B, Tran NL, Ryken T, Moore MK, Eqan KM, Olson JJ (2014). Current approaches to the treatment of metastatic brain tumours. Net Rev Clin Oncol.

[5] Bao Z, Liu X, Liu Y, Liu H, Zhao K (2016). Near-infrared light-responsive inorganic nanomaterials for photothermal therapy. Asian J Pharm Sci.

[6] Zhang Z, Wang J, Chen C (2013). Near-Infrared light-mediated nanoplatforms for cancer thermo-chemotherapy and optical imaging. Adv Mater.

[7] Chung US, Kim J, Kim B, Kim E, Jang W, Koh W (2016). Dendrimer porphyrin-coated gold nanoshells for the synergistic combination of photodynamic and photothermal therapy. Chem Commun.

[8] Dolmans DEJGJ, Fukumura D, Jain RK (2003). Photodynamic therapy for cancer. Nat Rev Cancer.

[9] Jaque D, Martinez Maestro L, del Rosal B, Haro-Gonzalez P, Benayas A, Plaza JL, Rodriguez EM, Sole JG (2014). Nanoparticles for photothermal therapies. Nanoscale.

[10] Chitgupi U, Qin Y, Lovell JF (2017). Targeted nanomaterials for phototherapy. Nanotheranostics.

[11] Zhang D, Wu M, Zeng Y, Wu L, Wang Q, Han X, Liu X, Liu J (2015). Chlorin e6 conjugated poly(dopamine) nanospheres as PDT/PTT dual-modal therapeutic agents for enhanced cancer therapy. ACS Appl Mater Interfaces.

[12] Hayashi K, Nakamura M, Miki H, Ozaki S, Abe M, Matsumoto T, Kori T, Ishimura K (2014). Nanoparticles with heavy-atom effect for wide-field photodynamic/photothermal therapy using single light source. Adv Funct Mater.

[13] Guo M, Mao H, Li Y, Zhu A, He H, Yang H, Wang Y, Tian X, Ge C, Peng Q, Wang X, Yang X, Chen X, Liu G, Chen H (2014). Dual imaging-guided photothermal/photodynamic therapy using micelles. Biomaterials.

[14] Huang X, Chen G, Pan J, Chen X, Huang N, Wang X, Liu J (2016). Effective PDT/PTT dual-modal phototherapeutic killing of pathogenic bacteria by using ruthenium nanoparticles. J Mater Chem B.

[15] Son KJ, Yoon HJ, Kim JH, Jang WD, Lee Y, Koh WG (2011). Photosensitizing hollow nanocapsules for combination cancer therapy. Angew Chem Int Ed.

[16] Topel SD, Cin GT, Akkaya EU (2014). Near IR excitation of heavy atom free Bodipy photosensitizers through the intermediacy of upconverting nanoparticles. Chem Commun.

[17] Sahu A, Choi WI, Lee JH, Tae G (2013). Graphene oxide mediated delivery of methylene blue for combined photodynamic and photothermal therapy. Biomaterials.

[18] Lee J, Lee YH, Choi JS, Park KS, Chang KS, Yoon M (2015). Hydrothermal synthesis of defective TiO_2_ nanoparticles for long-wavelength visible light-photocatalytic killing of cancer cells. RSC Adv.

[19] Carrillo-Torres RC, García-Soto MJ, Morales-Chávez SD, Garibay-Escobar A, Hernández-Paredes J, Guzman R, Barboza-Flores M, Álvarez-Ramos ME (2016). Hollow Au–Ag bimetallic nanoparticles with high photothermal stability. RSC Adv.

[20] Chandresekaran R, Lee AS, Yap LW, Jans DA, Wagstaff KM, Cheng W (2016). Tumor cell-specific photothermal killing by SELEX-derived DNA aptamer-targeted gold nanorods. Nanoscale.

[21] Ye X, Shi H, He X, Wang K, Li D, Qiu P (2014). Gold nanorod-seeded synthesis of Au@Ag/Au nanospheres with broad and intense near-infrared absorption for photothermal cancer therapy. J Mater Chem B.

[22] Abadeer NS, Murphy CJ (2016). Recent progress in cancer thermal therapy using gold nanoparticles. J Phys Chem C.

[23] Wang J, Zhu G, You M, Song E, Shukoor MI, Zhang K, Altman MB, Chen Y, Zhu Z, Huang CZ, Tan W (2012). Assembly of aptamer switch probes and photosensitizer on gold nanorods photodynamic cancer therapy. ACS Nano.

[24] Liao J, Li W, Peng J, Yang Q, Li H, Wei Y, Zhang X, Qian Z (2015). Combined cancer photothermal-chemotherapy based on doxorubicin/gold nanorod-loaded polymersomes. Theranostics.

[25] Kuo WS, Chang YT, Cho KC, Chiu KC, Lien CH, Yeh CS, Chen SJ (2012). Gold nanomaterials conjugated with indocyaine green for dual-modality photodynamic and photothermal therapy. Biomaterials.

[26] Kim JY, Choi WI, Kim M, Tae G (2013). Tumor-targeting nanogel that can function independently for both photodynamic and photothermal therapy and its synergy from the procedure of PDT followed by PTT. J Control Release.

[27] Jang B, Park JY, Tung CH, Kim IH, Choi Y (2011). Gold nanorod-photosensitizer complex for near-infrared fluorescence imaging and photodynamic/photothermal therapy in vivo. ACS Nano.

[28] Oh J, Yoon HJ, Park JH (2014). Plasmonic liposomes for synergistic photodynamic and photothermal therapy. J Mater Chem B.

[29] Menon JU, Jadeja P, Tambe P, Vu K, Yuan B, Nguyen KT (2013). Nanomaterials for photo-based diagnostic and therapeutic applications. Theranostics.

[30] Sakeye M, Smatt J (2012). Comparison of different amino-functionalization procedures on a selection of metal oxide microparticles: degree of modification and hydrolytic stability. Langmuir.

[31] Zhao J, Milanova M, Warmoeskerken MM, Dutschk V (2012). Surface modification of TiO_2_ nanoparticles with silane coupling agents. Colloids Surf A.

[32] Jokerst JV, Lobovkina T, Zare RN, Gambhir SS (2011). Nanoparticle PEGylation for imaging and therapy. Nanomedicine.

[33] Locatelli E, Monaco I, Franchini MC (2015). Surface modification of gold nanorods for applications in nanomedicine. RSC Adv.

[34] Schulz F, Friedrich W, Hoppe K, Vossmeyer T, Weller H, Lange H (2016). Effective PEGylation of gold nanorods. Nanoscale.

[35] Tatini F, Landini I, Scaletti F, Massai L, Centi S, Ratto F, Nobili S, Romano G, Fusi F, Messori L, Mini E, Pini R (2014). Size dependent biological profiles of PEGylated gold nanorods. J Mater Chem B.

[36] Manson J, Kumar D, Meenan BJ, Dixon D (2011). Polyethylene glycol functionalized gold nanoparticles: the influence of capping density on stability in various media. Gold Bull.

[37] Bartczak D, Kanaras AG (2011). Preparation of peptide-functionalized gold nanoparticles using one pot EDC/Sulfo-NHS coupling. Langmuir.

[38] Phong TQ, Phuong PTT (2016). Convalent conjugation of antibody and gold nanoparticle for development of lateral flow immunoassay test strip. J Sci Technol.

[39] Nomura T, Nakajima S, Kawabata K, Yamashita F, Takakura Y, Hashida M (1997). Intratumoral pharmacokinetics and in vivo gene expression of naked plasmid DNA and its cationic liposome complexes after direct gene transfer. Can Res.

[40] Frohlich E (2012). The role of surface charge in cellular uptake and cytotoxicity of medical nanoparticles. Int J Nanomed.

[41] Blanco E, Shen H, Ferrari M (2015). Principles of nanoparticle design for overcoming biological barriers to drug delivery. Nat Biotechnol.

[42] Panariti A, Miserocchi G, Rivolta I (2012). The effect of nanoparticle uptake on cellular behavior: disrupting or enabling functions?. Nanotechnol Sci Appl.

[43] Iversen T, Skotland T, Sandvig K (2011). Endocytosis and intracellular transport of nanoparticles: present knowledge and need for future studies. Nano Today.

[44] Zhu X, Fang C, Jia H, Huang Y, Cheng CHK, Ko C, Chen Z, Wang J, Wang YJ (2014). Cellular uptake behavior, photothermal therapy performance, and cytotoxicity of gold nanorods with various coatings. Nanoscale.

[45] You DG, Deepagan VG, Um W, Jeon S, Son S, Chang H, Yoon HI, Cho YW, Swierczewska M, Lee S, Pomper MG, Kwon IC, Kim K, Park JH (2016). ROS-generating TiO2 nanoparticles for non-invasive sonodynamic therapy of cancer. Sci Rep.

[46] Kang X, Guo X, Niu X, An W, Li S, Liu Z, Yang Y, Wang N, Jiang Q, Yan C, Wang H, Zhang Q (2017). Photothermal therapeutic application of gold nanorods-porphyrin-trastuzumab complexes in HER2-positive breast cancer. Sci Rep.

[47] Mamalis A, Koo E, Sckisel GD, Siegel DM, Jagdeo J (2016). Temperature-dependent impact of thermal aminoaevulinic acid photodynamic therapy on apoptosis and reactive oxygen species generation in human dermal fibroblasts. Br J Dermatol.

[48] Tsukamoto D, Shiraishi Y, Sugano Y, Ichikawa S, Tanaka S, Hirai T (2012). Gold nanoparticles located at the interface of anatase/rutile TiO_2_ particles as active plasmonic photocatalysts for aerobic oxidation. J Am Chem Soc.

[49] Furube A, Hashimoto S (2017). Insight into plamonic hot-electron transfer and plasmon molecular drive: new dimensions in energy conversion and nanofabrication. NPG Asia Mater.

[50] Sun H, Zeng S, He Q, She P, Xu K, Liu Z (2017). Spiky TiO_2_/Au nanorod plasmonic photocatalysts with enhanced visible-light photocatalytic activity. Dalton Trans.

[51] Yang Y, Wu J, Li J (2016). Correlation of the plasmon-enhanced photoconductance and photovoltaic properties of core-shell Au@TiO_2_ network. Appl Phys Lett.

[52] DuChene JS, Sweeny BC, Johnston-Peck AC, Su D, Stach EA, Wei WD (2014). Prolonged hot electron dynamics in plasmonic-metal/semiconductor heterostructures with implications for solar photocatalysis. Angew Chem Int Ed.

[53] Kodiyath R, Manikandan M, Liu L, Ramesh GV, Koyasu S, Miyauchi M, Sakuma Y, Tanabe T, Gunji T, Dao TD, Ueda S, Nagao T, Ye J, Abe H (2014). Visible-light photodecomposition of acetaldehyde by TiO_2_-coated gold nanocages: plasmon-mediated hot electron transport via defect states. Chem Commun.

