# Supplementary material for: Gold nanorods-conjugated TiO2 nanoclusters for the synergistic combination of phototherapeutic treatments of cancer cells
Source: J Nanobiotechnology. 2018 Dec 20;16:104. doi: 10.1186/s12951-018-0432-4 (PMC6300922; doi:10.1186/s12951-018-0432-4)
Supplement: Supplementary file 1 — Additional file 1: Figure S1. Sizes of APTES-TiO2 NPs (A), PEG-Au NRs (B) and Au NR-TiO2 NCs (C) were measured in deionized water at pH 7.0 by dynamic light scattering (DLS). Figure S2. Surface charge or zeta potential of TiO2 NPs (A), Au NRs (B) and Au NR-TiO2 NCs (C) were measured in deionized water at pH 7.0 by laser doppler velocimetry (LDV). Figure S3. Laser scanning confocal microscopy images of HeLa cells. DIC images and fluorescence images indicating ROS generation with nanoparticles in the dark (A) or under NIR (808 nm laser) light irradiation (B). Figure S4. Temperature changes of the cell-culture solutions containing various concentrations of APTES-TiO2 NPs (A), PEG-Au NRs (B) and Au NR-TiO2 NCs (C) with various as a function of NIR (808 nm laser) exposure time. Figure S5. Schematic illustration of the optical system for light irradiation. [file 12951_2018_432_MOESM1_ESM.docx]

**Additional file**

Gold nanorods-conjugated TiO_2_ nanocomplexes for the synergistic combination of phototherapeutic treatments of cancer cells

Jooran Lee,*^a,c^* Young Hwa Lee,*^b^* Chan Bae Jeong,*^a^* Joon Sig Choi,*^b^* Ki Soo Chang, **^a^* and Minjoong Yoon *^d^

1. Division of Scientific Instrumentation, Korea Basic Science Institute, Daejeon 34133, Republic of Korea
2. Department of Biochemistry, College of Natural Sciences, Chungnam National University, Daejeon 34134, Republic of Korea
3. Medical Device Development Center, Osong Medical Innovation Foundation, Cheongju, Chungbuk 28160, Republic of Korea
4. Department of Chemistry, College of Natural Sciences, Chungnam National University, Daejeon 34134, Republic of Korea. E-mail: mjyoon@cnu.ac.kr


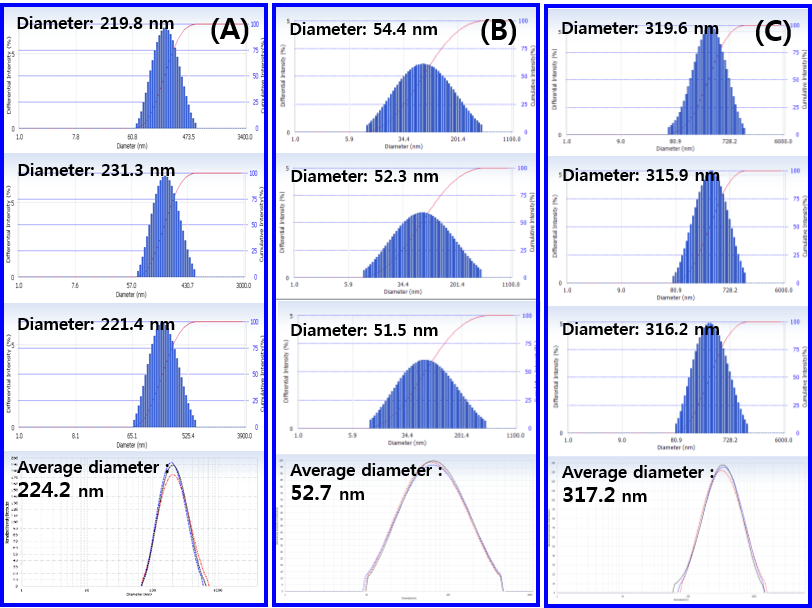


Figure S1. Sizes of APTES-TiO_2_ NPs (A), PEG-Au NRs (B) and Au NR-TiO_2_ NCs (C) were measured in deionized water at pH 7.0 by dynamic light scattering (DLS).


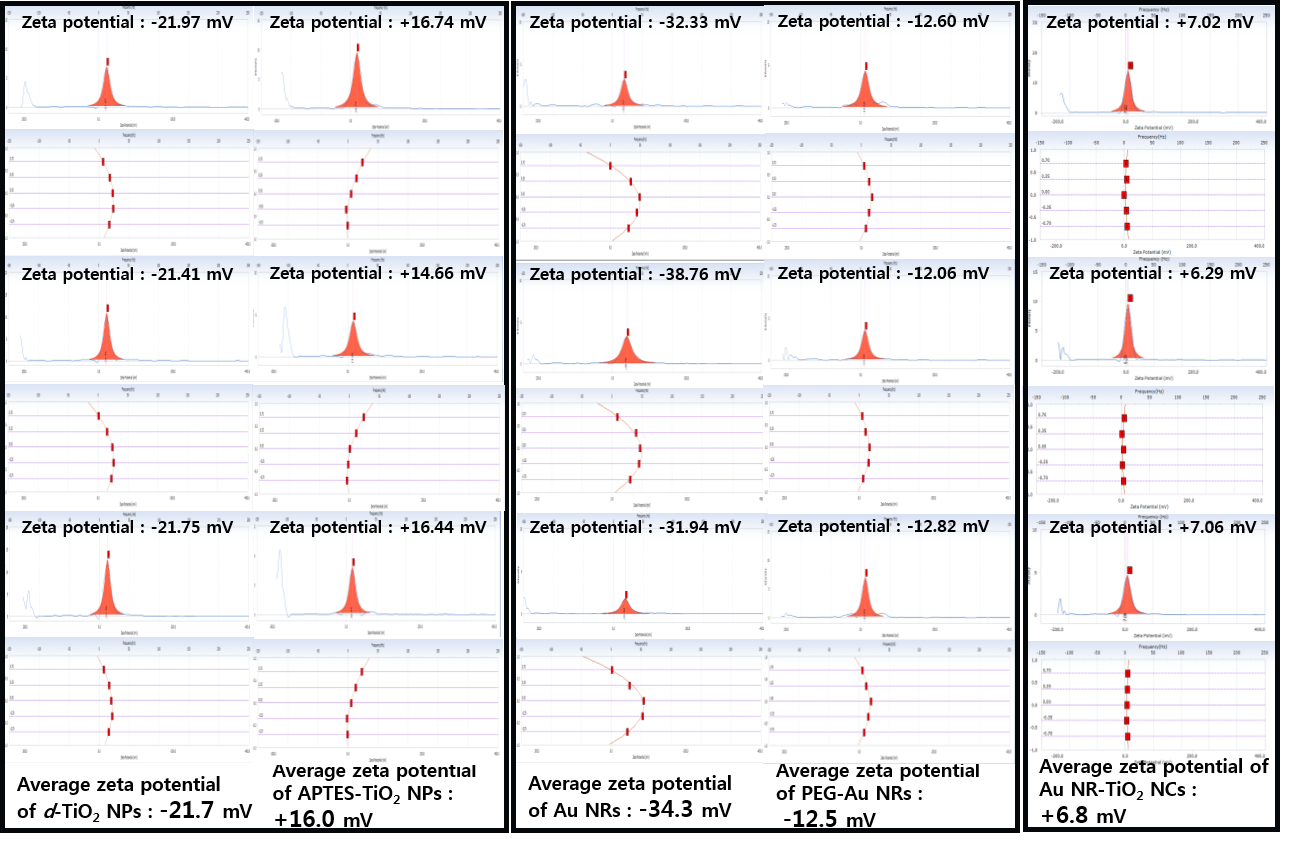


Figure S2. Surface charge or zeta potential of TiO_2_ NPs (A), Au NRs (B) and Au NR-TiO_2_ NCs (C) were measured in deionized water at pH 7.0 by laser doppler velocimetry (LDV).


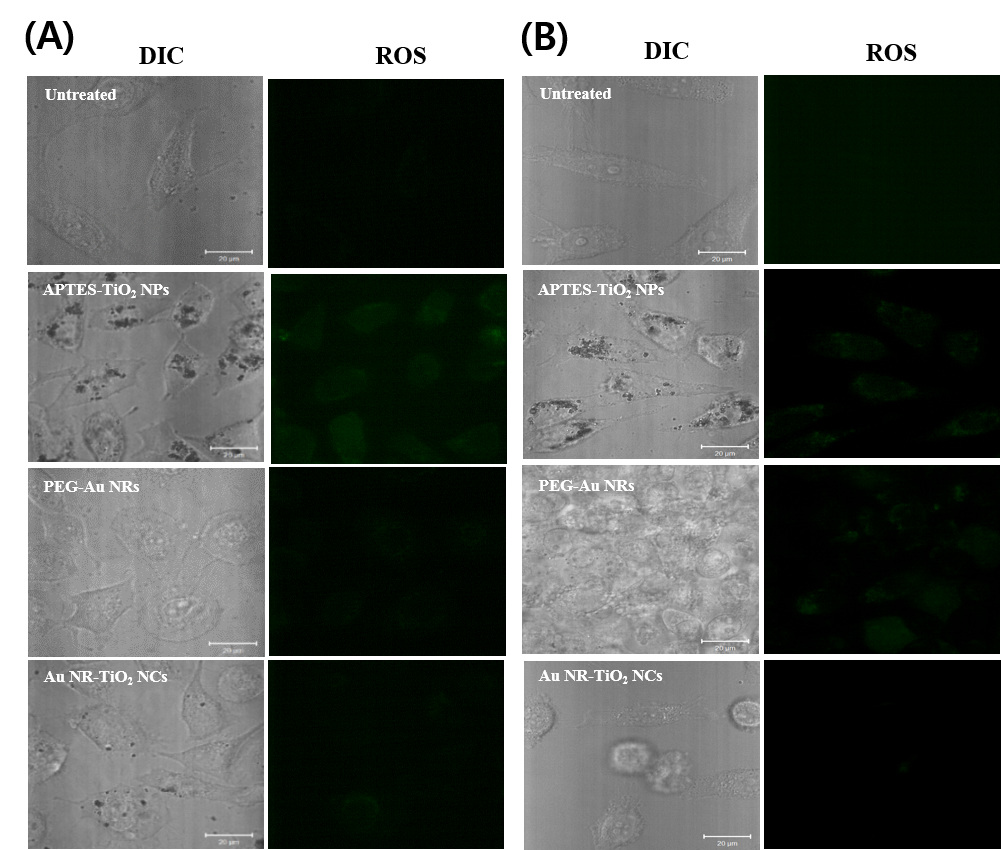


Figure S3. Laser scanning confocal microscopy images of HeLa cells. DIC images and fluorescence images indicating ROS generation with nanoparticles in the dark (A) or under NIR (808 nm laser) light irradiation (B).


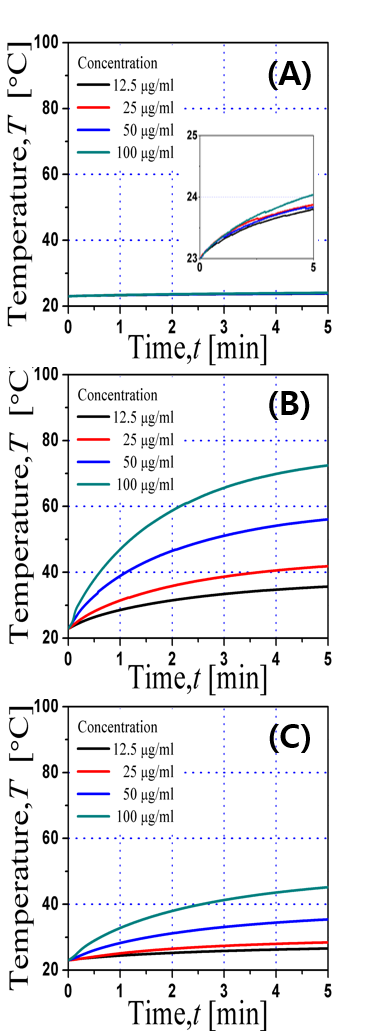


Figure S4. Temperature change of the cell culture solution containing APTES-TiO_2_ NPs (A), PEG-Au NRs (B) and Au NR-TiO_2_ NCs (C) with various concentrations of nanoparticles as a function of NIR (808 nm laser) exposure time.


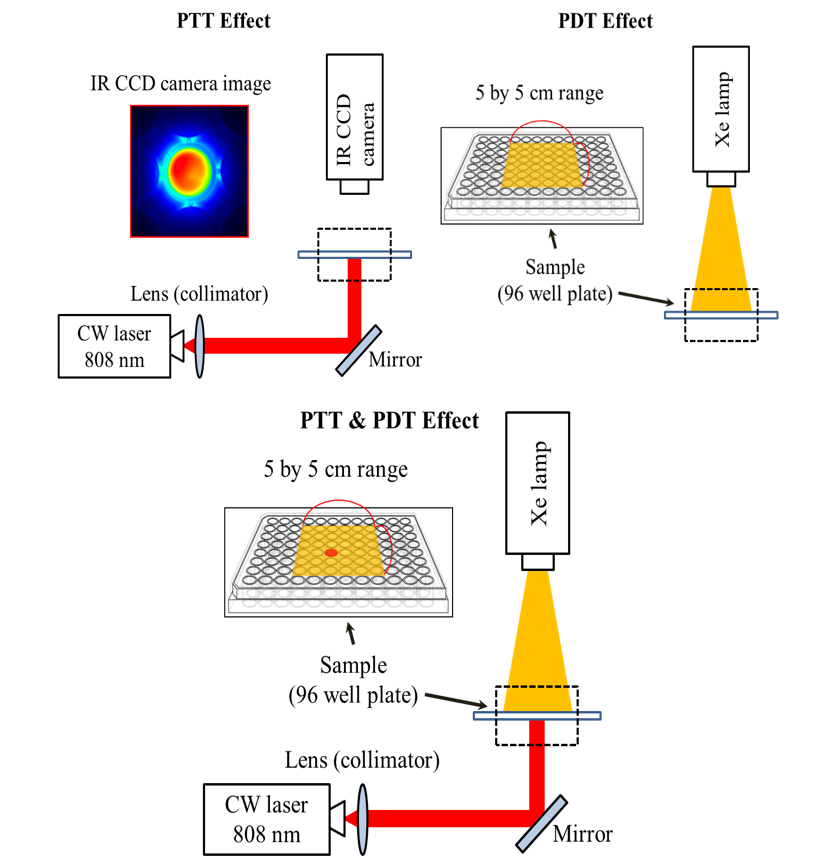


Figure S5. Schematic illustration of the experimental set-up.
